# Pairing Spruce Budworm Control and Minimal Understory Perturbations: Effects of Btk Spraying Frequency in Boreal Forests

**DOI:** 10.1002/ece3.73188

**Published:** 2026-02-27

**Authors:** Mathilde Robitaille, David Pothier, Stéphanie Pellerin

**Affiliations:** ^1^ Institut de Recherche en Biologie Végétale, Département de Sciences Biologiques Université de Montréal Montréal Canada; ^2^ Département Des Sciences du Bois et de la Forêt Université Laval Québec Canada; ^3^ Jardin Botanique de Montréal Montréal Canada

**Keywords:** arboreal lichens, *Bacillus thuringiensis*, spray operations, spruce budworm, understory vegetation

## Abstract

Since 1985, more than 10 million hectares of Canadian forests have been treated against spruce budworm (SBW) epidemics using the Btk biological insecticide. Still, no study has thoroughly evaluated the effects of these interventions on understory vegetation. Since the forest floor hosts most of the plant diversity in boreal forests and provides critical habitats for wildlife, it is crucial to determine the best treatment to reduce defoliation caused by SBW, while minimizing disturbances to understory plant communities. Using an experimental design established in 2007, we tested in 2022 the effects of different Btk spraying frequencies on tree defoliation, understory vegetation, and arboreal lichens. Our results show that reducing the spraying frequency increased light availability in the understory, leading to higher vascular plant richness, increased cover of shrubs, forbs, and shade‐intolerant species, and a greater number of fruits produced by fleshy‐fruit bearing plants, while arboreal lichen biomass remained unchanged. Although annual spraying most effectively limits SBW‐induced defoliation, such an approach may not be necessary to maintain canopy closure. Biennial applications appear sufficient to preserve foliage during SBW infestation, while limiting understory shifts toward early‐successional vegetation.

## Graphical abstract

Spruce budworm epidemic induces tree mortality and modifies understory communities. Could aerial spraying of Btk reduce perturbations in understory communities? Biannual Btk spraying could prevent both wood loss and understory degradation.

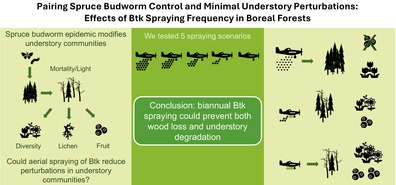

## 1 Introduction

In the eastern North American boreal forest, the spruce budworm ( *Choristoneura fumiferana* ; SBW) is the most damaging outbreaking insect (Montoro Girona et al. 2018) and the primary agent of secondary disturbances (Martin et al. 2019). SBW outbreaks occur every ca. 30 years (Martin et al. 2019; Morin et al. 2009), resulting in defoliation of the SBW‐preferred tree hosts (mainly *Abies balsamea* but also *Picea* spp.). *Abies balsamea* mortality following defoliation can vary between 33% and 99% depending on stand composition, number of consecutive years with severe defoliation, and stand age (Bouchard et al. 2005).

Tree defoliation and mortality induce changes in canopy openness (D'Aoust et al. 2004), which can alter understory composition and stand successional dynamics (Chagnon et al. 2022; Kneeshaw and Bergeron 1998; Leduc et al. 2020). For example, high canopy openness following SBW outbreaks favors the cover of shrubs, ferns, forbs, and fruit‐bearing species but is detrimental to terricolous lichen and ericaceous shrub covers (Chagnon et al. 2022). Concerning tree regeneration, canopy openness mainly benefits shade‐intolerant deciduous species, which can become dominant in the canopy decades following the outbreak (Chagnon et al. 2022; D'Aoust et al. 2004). SBW outbreaks can also promote local habitat heterogeneity and foster plant diversity (Crispo et al. 2021), especially at an intermediate level of disturbance (Taylor and Chen 2011). At such a level of disturbance, most shade‐tolerant species can persist in microsites following canopy gap creation that, in turn, favors the establishment of shade‐intolerant species (de Grandpré and Bergeron 1997). Furthermore, SBW‐induced defoliation may indirectly affect the abundance of arboreal and terrestrial lichens by promoting tree mortality and the establishment of fast‐growing deciduous species (Chagnon et al. 2022; Rominger and Oldemeyer 2011; Stone et al. 2008; Thompson et al. 2015). Large, living trees usually support more lichens than dead or small trees (Campbell and Coxson 2001; Waterhouse et al. 2007), while lichens are often less abundant in deciduous stands due to low light availability and high litter inputs (Joly et al. 2010).

Due to extensive tree mortality and the resulting economic losses that SBW outbreaks can cause (Chang et al. 2012; Hennigar et al. 2013), forest managers in Canada often try to reduce the impacts of these epidemics on stand foliage using a *Bacillus thuringiensis* ssp. *kurstaki* (Btk) insecticide formulation (van Frankenhuyzen et al. 2016). Btk is a soil bacterium that produces a proteinaceous crystal specifically toxic to *Lepidoptera*. When ingested, the crystal provokes gut damage, leading to the insect's death (Höfte and Whiteley 1989; van Frankenhuyzen et al. 2016). This insecticide has been sprayed over about 10 million ha of forest in Canada since its first sprayings against the SBW in 1985 (van Frankenhuyzen et al. 2016). In Quebec, the current Btk spraying strategy involves applications when moderate to severe defoliation is observed to maintain current‐year defoliation under a 50% threshold and ensure low *A. balsamea* mortality (Clark 1961; Hardy and Dorais 1976). This strategy effectively protects tree foliage (Fuentealba et al. 2019; Liu et al. 2020), but the understory response to outbreak control is still poorly known. Since the forest floor hosts most plant diversity, plays a critical role in nutrient cycling and net productivity, and provides critical habitats for wildlife, including the endangered woodland caribou (*Rangifer tarandus caribou*) (de Grandpré et al. 2022; Kumar et al. 2018; Nilsson and Wardle 2005), it is crucial to determine the best Btk spraying frequency to reduce tree defoliation, application costs and disturbances of understory communities. Furthermore, how SBW epidemic controls could affect the abundance of arboreal lichens remains to be evaluated.

This study aimed to determine the effects of a gradient of five Btk aerial spraying frequencies on SBW‐induced tree defoliation, understory vegetation, and arboreal lichen biomass using an experimental design established in 2007. We predicted that (1) less frequent Btk sprayings would increase light availability in the understory through defoliation and mortality of *A. balsamea* and *Picea* spp. trees (D'Aoust et al. 2004), (2) understory diversity would be maximal at intermediate Btk spraying frequencies due to the persistence of shade‐tolerant species and the establishment of pioneer, shade‐intolerant plants (de Grandpré and Bergeron 1997; Taylor and Chen 2011), and (3) the proportion of shade‐intolerant species, the abundance of deciduous and fruit‐bearing shrubs, and the number of fruits produced would increase with decreasing Btk spraying frequency, while the biomass of arboreal lichens would decrease with decreasing Btk spraying frequency (Chagnon et al. 2022; D'Aoust et al. 2004; Waterhouse et al. 2007). Our predictions assume that understory light availability is a primary driver of plant recruitment, persistence, and fitness following SBW outbreaks and controls.

## 2 Methods

### 2.1 Study Area

**FIGURE 1:**
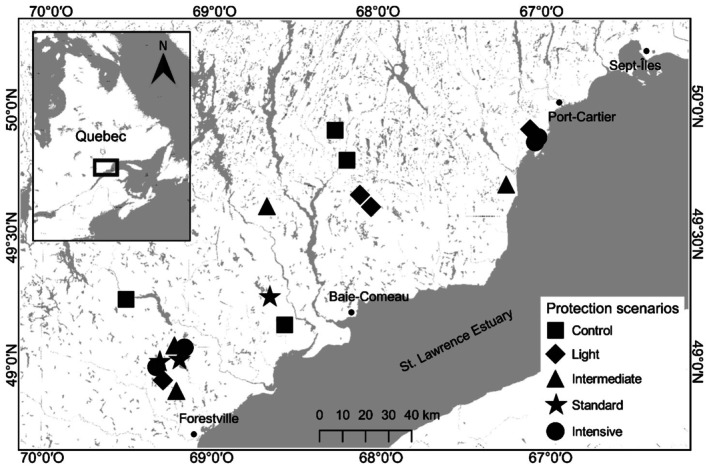
Study area and location of the 19 experimental units sampled in 2022 used to assess differences in stand and understory composition of forest stands spraying with different Btk application frequencies (protection scenarios) since 2007. Gray areas correspond to water surfaces.

The study area is located on the North Shore of the St. Lawrence Estuary in eastern Quebec, Canada (Figure 1). The mean annual temperature fluctuates between 1.2°C at Sept‐Îles and 2.1°C at Baie‐Comeau meteorological stations (Canadian Climate Normals 1991–2020 Data; Environment Canada 2025), with warmer temperatures observed along the coast of the estuary (Régnière and St‐Amant 2007). Total annual precipitation ranges from 966 mm in the southwest (Baie‐Comeau) to 1077 mm in the northeast (Sept‐Îles) of the study area. The region is part of the Canadian Shield, primarily consisting of Precambrian rocks covered by glacial tills and fluvioglacial deposits (Bouchard, Kneeshaw, and Bergeron 2008). Vegetation belongs to the boreal forest, particularly the Eastern *Abies balsamea* ‐ *Betula papyrifera* and the Eastern *Picea mariana* ‐mosses bio‐climatic sub‐domains (Saucier et al. 2011). On mesic sites, forest stands are mainly composed of *A. balsamea* and *P. mariana* , with the presence of *Pinus banksiana* , *B. papyrifera* , *Populus tremuloides* , and, in the southernmost plots, *Acer rubrum* . Understory vegetation is mainly composed of native geophytes (e.g., *Maianthemum canadense* , *Clintonia borealis* , *Aralia nudicaulis* ) and shrubs (e.g., *Kalmia angustifolia, Acer spicatum*, *Taxus canadensis* ), while the forest floor is dominated by mosses, mainly *Hylocomium splendens* and *Pleurozium schreberi* (de Grandpré et al. 2011; Fourrier et al. 2015).

Historically, the region experienced minor SBW outbreaks until 1980, when a severe outbreak caused extensive stand mortality (Berguet et al. 2021; Bouchard and Pothier 2010). The current outbreak, which began around 2006 (Cotton‐Gagnon et al. 2018), affected 13.5 million hectares at its peak in 2020 (MRNF 2020), and still covered 910,723 ha in 2024 (MRNF 2024). Other natural disturbances in the region include fire and windthrow. The mean fire return interval is about 270 years, although it can reach 500 years in the eastern part of the study area (Bouchard, Pothier, and Gauthier 2008). Since 1800, some extensive fires, notably two events covering approximately 1500 km^2^ in 1921 and 1991, have impacted stand composition (Bouchard, Pothier, and Gauthier 2008; Bouchard and Pothier 2011). Stand‐replacing windthrows, characterized by severe mortality over an area exceeding 5 ha, occur relatively infrequently in the region, corresponding to a cycle of 3900 years (Bouchard et al. 2009). Nevertheless, smaller windthrow events, which may not necessarily result in stand replacement, are critical in boreal forest dynamics, creating gaps in the canopy structure and promoting the growth of smaller trees (Bouchard et al. 2009; Martin et al. 2019). Logging is the main human disturbance. At the beginning of the 1900s, it was a minor disturbance as the industry was mostly restricted to selective logging, but it gained importance in the early 1920s with the advent of industrial forestry and clearcutting practices (Bouchard and Pothier 2011). Logging exerts a notable influence on stand composition and the age structure of the forest landscape (Boucher and Grondin 2012), although unmanaged forest stands still cover about 28% of the study area, mostly in the northeast (MRNF 2025).

### 2.2 Experimental Design

**FIGURE 2:**
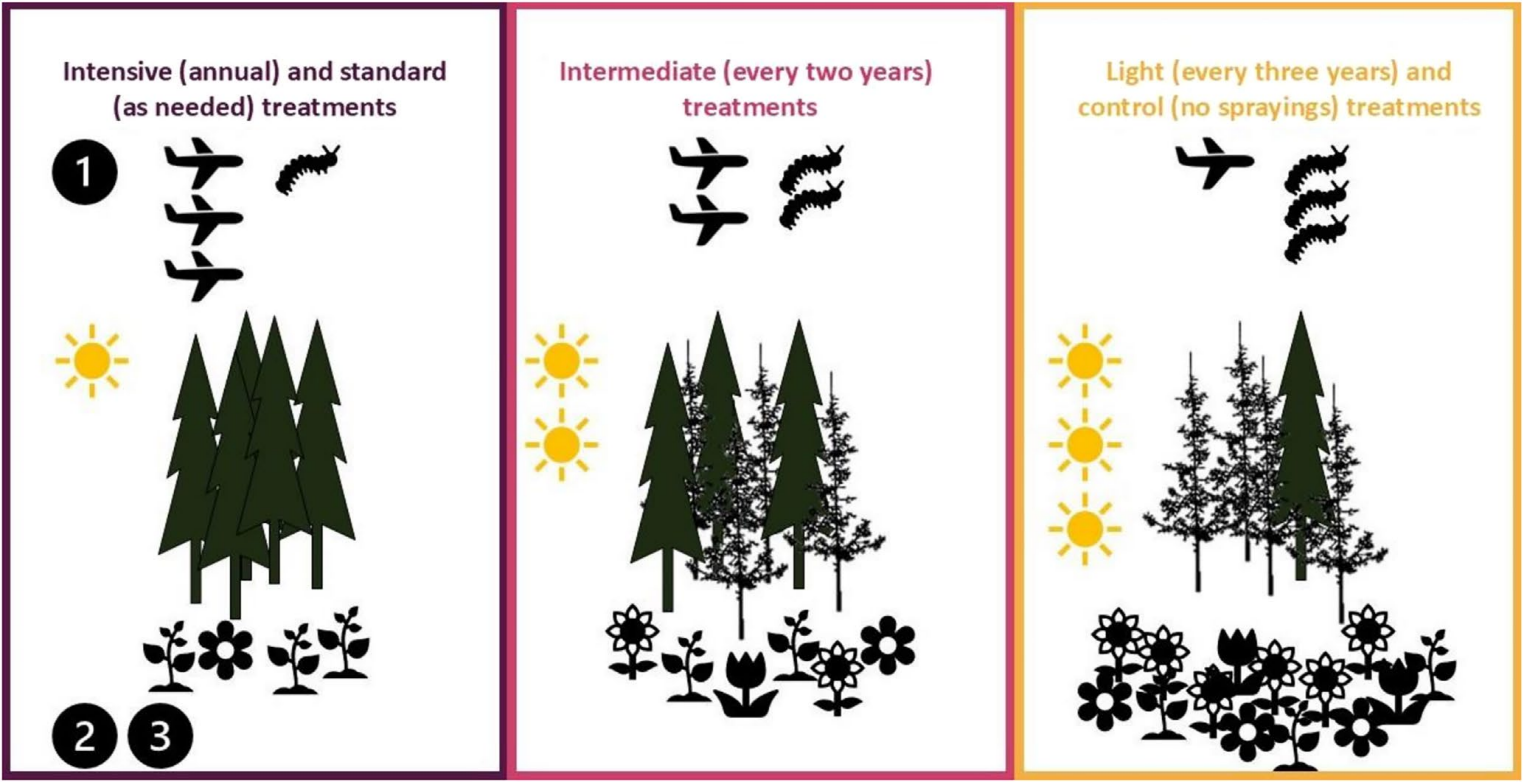
Schematic representation of the Btk treatments tested in this study and their hypothetical effects on foliage cover and understory vegetation. The numbers in the figure represent the different predictions (1. light availability, 2. and 3. understory diversity and understory composition).

We collected data in an incomplete randomized experimental design (Figure 1) established in 2007 by the *Société de protection des forêts contre les insectes et les maladies* (SOPFIM; Fuentealba et al. 2019). In this design, twenty ca. 100‐ha experimental units were established according to the presence of SBW, a high degree of stand vulnerability (> 75% of the stand is composed of *A. balsamea* ), and accessibility. Each experimental unit was assigned to one of the five Btk protection scenarios tested (Figure 2), with four replicates per scenario. The five Btk protection scenarios corresponded to intensive protection (annual Btk spraying), standard protection (spraying as needed to protect 50% of the current year's foliage, which resulted in spraying in 11 of the 13 years of defoliation), intermediate protection (spraying every 2 years), light protection (spraying every 3 years), and control plots (no spraying). We excluded one experimental unit belonging to the standard protection scenario because SBW populations in this area were lower than expected. The standard protection scenario served as a reference as it reflects the strategy currently used in Quebec. It corresponds to approximately 60% of the sprayings applied in the intensive protection scenario. Aerial sprayings of the commercial insecticide Btk were conducted by SOPFIM early in the morning or at dusk on days without rain or significant wind. Btk spraying was timed to coincide with the flushing of balsam fir shoots, which provides optimal stand protection (Carisey et al. 2004). For further details on the experimental design and Btk formulation and application, see Fuentealba et al. (2019). For data collection, three 400‐m^2^ plots (20 × 20 m) were established in each experimental unit (57 plots) (Figure S1).

#### 2.2.1 Plant Area Index

Plant area index (PAI) quantifies the plant material in a stand. It can be determined using hemispherical photography and is an easy way to estimate cumulative defoliation (Donovan et al. 2018) and therefore, light availability in the understory. We took all hemispherical photos in June 2022 using a Panasonic Lumix DMC‐LX5GK camera and a Nikon FC‐E8 circular fisheye lens mounted on a self‐leveling Mid‐O‐Mount support (Regent Instruments Inc., Quebec, Canada) and a tripod with bubble level. We avoided as much as possible bright conditions such as under a clear blue sky or a direct sunbeam. We positioned and leveled the camera at 1.3 m above ground and oriented it so that the top of the image was directed north. Within each 400‐m^2^ plot, we took 12 hemispherical photos, i.e., three photos taken at different exposure levels (−1, 0, 1, to avoid exposure determination issues during photo analysis) at 6 m from the center of the plot at each of the four cardinal points (Figure S1). We retained only the photos with the best exposure for analysis, for a total of 228 photos (resolution = 2736 × 2736 pixels).

We analyzed hemispherical photos using WinSCANOPY software (v. 2016a Pro, Regent Instruments Inc., Quebec, Canada). We applied circular masking to the images to analyze only zenith angles from 0° to 60°. When necessary, we edited photos to remove image defects and prevent incorrect pixel classification. We determined pixel classification between plant material and sky based on gray levels. We used automatic thresholding whenever possible, but manual threshold adjustment was often necessary for photos taken under bright conditions. We calculated PAI with WinSCANOPY software, which uses LI‐COR's LAI‐2000 Generalized log method using the sky grid's zenith rings and Lang and Yueqin (1986) correction for clumping compensation.

#### 2.2.2 Arboreal Lichens Sampling

To sample arboreal lichens, we selected the four living trees ( *A. balsamea* or *Picea* spp.) closest to the center of each 400‐m^2^ plot, with a diameter at breast height (DBH) greater than 9 cm. We estimated the biomass of the three most common arboreal lichen genera in the region, namely *Bryoria*, *Evernia*, and *Usnea*, on the trunks and branches of each selected tree, between one and three meters above the ground (Boudreault et al. 2015). We estimated biomass using the clump method, which involves visually comparing lichen accumulations on each tree to standard reference clumps of known dry mass for each lichen genus (Campbell et al. 1999). Three reference clumps (one per genus), oven‐dried for 24 h at 60°C and weighed to the nearest 0.001 g, were used as references. For each tree, we counted the number of reference units present and multiplied this number by the weight of the reference unit to estimate the biomass (Boudreault et al. 2015). To ensure consistency, estimations were validated by two observers. We summed the biomass estimated for the three lichen genera for each tree. Since no significant relationships were found between lichen biomass and tree DBH (Figure S2), we calculated the total lichen biomass (kg per hectare) by multiplying the mean biomass value of the four trees sampled by the number of living *A. balsamea* and *Picea* spp. trees per plot, which was then transformed per hectare.

#### 2.2.3 Understory Plant Communities Sampling

We sampled understory plant communities between June 26th and July 13th, 2022, within each 400‐m^2^ plot using 12 square 1‐m^2^ subplots. We established subplots at 3, 6, and 9 m from the center of each experimental unit along transects stretching out in the four cardinal directions (*n* = 684 subplots) (Figure S1). In each subplot, we visually estimated the cover of each plant species, including tree seedlings (height ≤ 1.5 m) but excluding saplings (height > 1.5 m and DBH < 9 cm) and trees using seven cover classes: < 1%, 1%–5%, 6%–25%, 26%–50%, 51%–75%, 76%–95%, 96%–100%. For statistical analyses, we used the median of the cover classes. Nomenclature of plant species follows VASCAN (Brouillet et al. 2010).

In August 2022, we revisited each of the 12 1‐m^2^ subplots in which fleshy‐fruit bearing species (*Amelanchier* spp., *Rubus idaeus* , *Vaccinium angustifolium* or *V. myrtilloides* ) were identified to count the number of fruits produced. Counts were limited to 1 min and, if needed, the number of fruits produced was extrapolated for the other plants of that species according to Larsen et al. (2019). The number of fruits for each site was then transformed into the number of fruits/m^2^.

### 2.3 Statistical Analyses

To test whether there was a significant difference in stand PAI among Btk protection scenarios (Prediction 1), we built a linear mixed‐effect model (see Table S1 for detail description of all models) using hemispherical photos as entities (228 entities: 4 photos × 57 plots). Experimental units were considered as a random factor, which allowed us to control for similarities among plots located in the same experimental unit. As residuals did not follow a normal distribution, we log‐transformed the PAI data.

To test whether vascular plant alpha diversity (Prediction 2) and arboreal lichens biomass (Prediction 3) differed between Btk protection scenarios, we used the same linear mixed‐effect model procedure described above. We tested two measures of vascular plant alpha diversity: species richness and Shannon Weaver Index (Shannon 1948). We calculated species richness as the number of distinct vascular species in each plot. For the calculation of the Shannon Weaver Index, we used the mean cover of each species as a surrogate of abundance (Upton 2022). We calculated the mean cover of each species in each 400‐m^2^ plot by averaging data of the 12 subplots. We log‐transformed the lichen biomass data as residuals did not follow a normal distribution. The effects of the treatments on terrestrial lichens were not tested because they occurred in fewer than 1% of the sampled plots (mean cover of 0.07%).

To evaluate whether some groups of species were favored by spraying frequency (Prediction 3), we compared the Importance Value (IV) of life‐form groups (deciduous trees, coniferous trees, shrubs, forbs, ferns/allies, terrestrial lichens and bryophytes) and shade tolerance groups between Btk protection scenarios (Table S1). The IV indicates the degree of dominance of a species or group of species within a community and enables the comparison of sites with different absolute percent covers (Barbour et al. 1980). Life‐form groups followed Brouillet et al. (2010) and species shade tolerance follows the Traits of Plants In Canada database (TOPIC, Aubin et al. 2012), Humbert et al. (2007) as well as USDA and NRCS (2015). For species identified at the genus level, we estimated shade tolerance based on species likely to occur in the study area based on occurrence data in Canadensys (canadensys.net). For each 400‐m^2^ plot, we calculated the IV of each group of plants by summing the cover of all species within each group and then dividing this cover by the cover of all species. We used a linear mixed‐effect model to compare IV and Btk protection scenarios among species groups (life‐form or shade tolerance), and to test the interaction between both factors. We considered the experimental unit as a random factor. We arcsin‐transformed the IV values for life‐form groups as residuals did not follow a normal distribution. Finaly, we identified indicator species of Btk protection scenarios using the IndVal index (Prediction 3) (Dufrêne and Legendre 1997). This index is the product of the degree of specificity (uniqueness to a particular group) and the degree of fidelity (frequency of occurrence within a particular group) of species in groups defined a priori, here by the Btk protection scenarios.

To test whether vascular plant beta diversity differed between Btk protection scenarios (Prediction 2), we used the distance‐based test for the homogeneity of multivariate dispersions (PERMDISP; Anderson et al. 2006). PERMDISP calculates the distance of each site (here each 400‐m^2^ plot) to the centroid in an ordination space (principal coordinates analysis) and then tests whether these distances are different between groups (spraying scenarios) through permutation tests. We first built a site‐by‐site distance matrix on a species presence‐absence matrix using Hellinger distances to compute the centroid of each group (Legendre and Gallagher 2001). Then, we calculated the distance of each site to its associated group centroid, and the dispersion of these distances (within‐group variance) was used as an estimate of beta diversity (the greater the within‐group variance, the higher the beta diversity). Finally, we tested for differences in the average site‐to‐centroid distances of groups (variance) by performing an analysis of variance with 9999 permutations. To detect shifts in species composition between groups, we tested for location differences between centroids (the greater the distance between centroids, the greater the differences in species composition) using multivariate analysis of variance (PERMANOVA; Anderson 2001) with 9999 permutations.

For every linear model described above, as well as mixed effects models, we also tested models including environmental variables (e.g., distance to the coast, surface deposit, mean temperature; Table S2) and canopy openness (PAI) to explain the variation in alpha diversity, lichen biomass and shade tolerance and life‐form IV, but the results were either non‐significant or less parsimonious (Robitaille 2024).

We performed all analyses in the R environment (v. 4.2.1; R Core Team 2022) using *lme4* (v. 1.‐1‐31; Bates et al. 2015; fitting linear mixed models), *lmerTest* (v. 3.1‐3; Kuznetsova et al. 2017; *p*‐value for linear mixed models), *car* (v. 3.1‐1; J. Fox and Weisberg 2019; anova tables for linear models), *emmeans* (v. 1.8.5; Russell et al. 2023; post hoc tests on linear mixed models) *indicspecies* (v. 1.7.15; de Cáceres and Legendre 2009; indicator species), and *vegan* (v. 2.6‐4; Oksanen et al. 2022; alpha and beta diversity computation).

## 3 Results

### 3.1 Relationship Between Btk Protection Scenarios and Stand PAI

**FIGURE 3:**
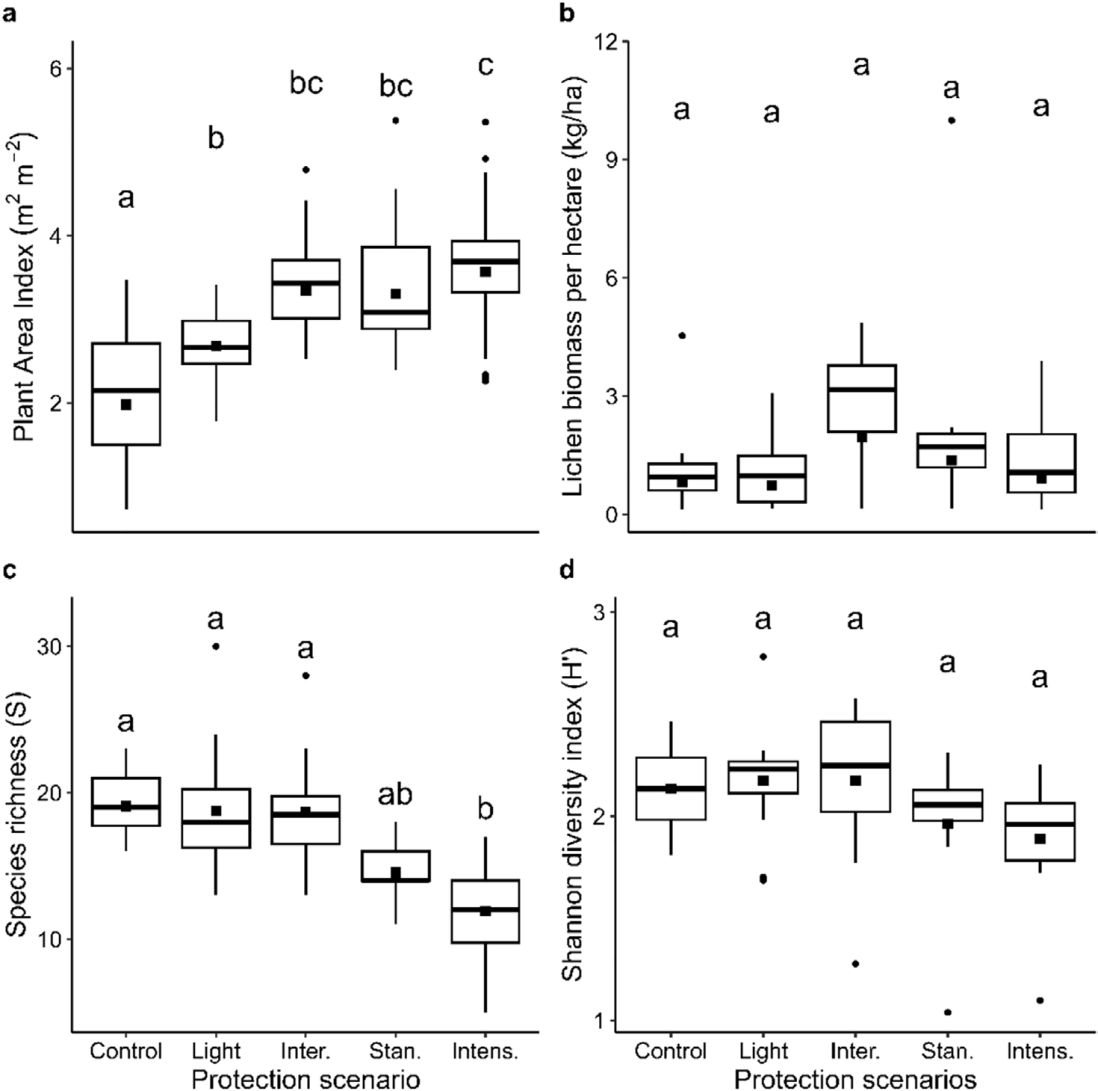
Influence of Btk protection scenarios that have been applied in northeastern Québec since 2007 on (a) plant Area Index, (b) arboreal lichen biomass per hectare, (c) species richness, and (d) Shannon diversity index calculated with 2022 data. Black square: Mean; bold horizontal line: Median; boxes: 25%–75% quartiles; whisker: Ranges; black dot: Outliers. Different lower‐case letters indicate a significant difference between protection scenarios (*p* < 0.05).

The average PAI calculated across all 19 experimental units was 3.02 m^2^ m^−2^ (i.e., 3.02 m^2^ of plant material by m^2^ of ground area), and values ranged from 0.72 m^2^ m^−2^ (control plot) to 5.38 m^2^ m^−2^ (standard protection scenario) (Figure 3a). We observed significant differences in PAI among Btk protection scenarios (*F*_(4,52)_ = [14.516], *p* < 0.001). Mean PAI was significantly lower in control plots compared to all other protection scenarios. In addition, the PAI of plots under the light protection scenario was significantly lower than in plots under the intensive protection scenario but was similar to plots under intermediate and standard protection scenarios. Overall, PAI decreased with decreasing spraying frequency.

### 3.2 Relationship Between Btk Protection Scenarios and Arboreal Lichen Biomass and Understory Vegetation

The average lichen biomass per hectare across all 19 experimental units was 1.84 kg/ha, and values ranged from 0.16 kg/ha to 10.1 kg/ha (Figure 3b). There were no significant differences between protection scenarios (*F*_(4,14)_ = [1.1577], *p* = 0.37).

We identified 67 vascular plant species in the 19 experimental units. The average richness per 400 m^2^ plot was 17 species and values ranged from 5 to 30 species (Figure 3c). Richness was significantly higher in control plots and plots of light and intermediate protection scenarios than in plots of intensive protection (*F*_(4,14)_ = [4.733], *p* = 0.01). Shannon diversity index varied between 1.04 and 2.78 and was not significantly different between protection scenarios (*F*_(4,14)_ = [1.032], *p* = 0.42, Figure 3d). We identified 8 significant indicator species (Table 1). Four species ( *Rubus idaeus* , *Chamerion angustifolium* subsp. *angustifolium*, *Viburnum cassinoides* , and *Dryopteris cristata* ) were indicators of plots under the control scenario, two species ( *Populus balsamifera* and *Rubus pubescens* ) of plots under the light scenario, and two species ( *Eurybia macrophylla* and *Taxus canadensis* ) of plots under the intermediate scenario. Among these species, *Rubus idaeus* and *Taxus canadensis* were those with the highest average cover (Table S3).

**TABLE 1:** List of plant indicator species identified with 2022 data for each Btk protection scenario have been applied in northeastern Québec since 2007 with their respective specificity and fidelity values, IndVal percentage and *p*‐value. No species were found to be indicator of standard and intensive scenarios.

Protection scenarios	Species	Specificity	Fidelity	IndVal	*p*
Control	*Rubus ideaus*	0.9	0.6	0.725	0.001
	*Chamerion angustifolium*	0.6	0.4	0.512	0.047
	*Viburnum cassinoides*	1.0	0.3	0.500	0.026
	*Dryopteris cristata*	0.9	0.3	0.497	0.043
Light	*Populus balsamifera*	1.0	0.5	0.500	0.040
	*Rubus pubescens*	0.9	0.5	0.498	0.045
Intermediate	*Eurybia macrophylla*	0.8	0.6	0.606	0.009
	*Taxus canadensis*	0.8	0.5	0.584	0.005
Standard		—	—	—	—
Intensive	—	—	—	—	—

**FIGURE 4:**
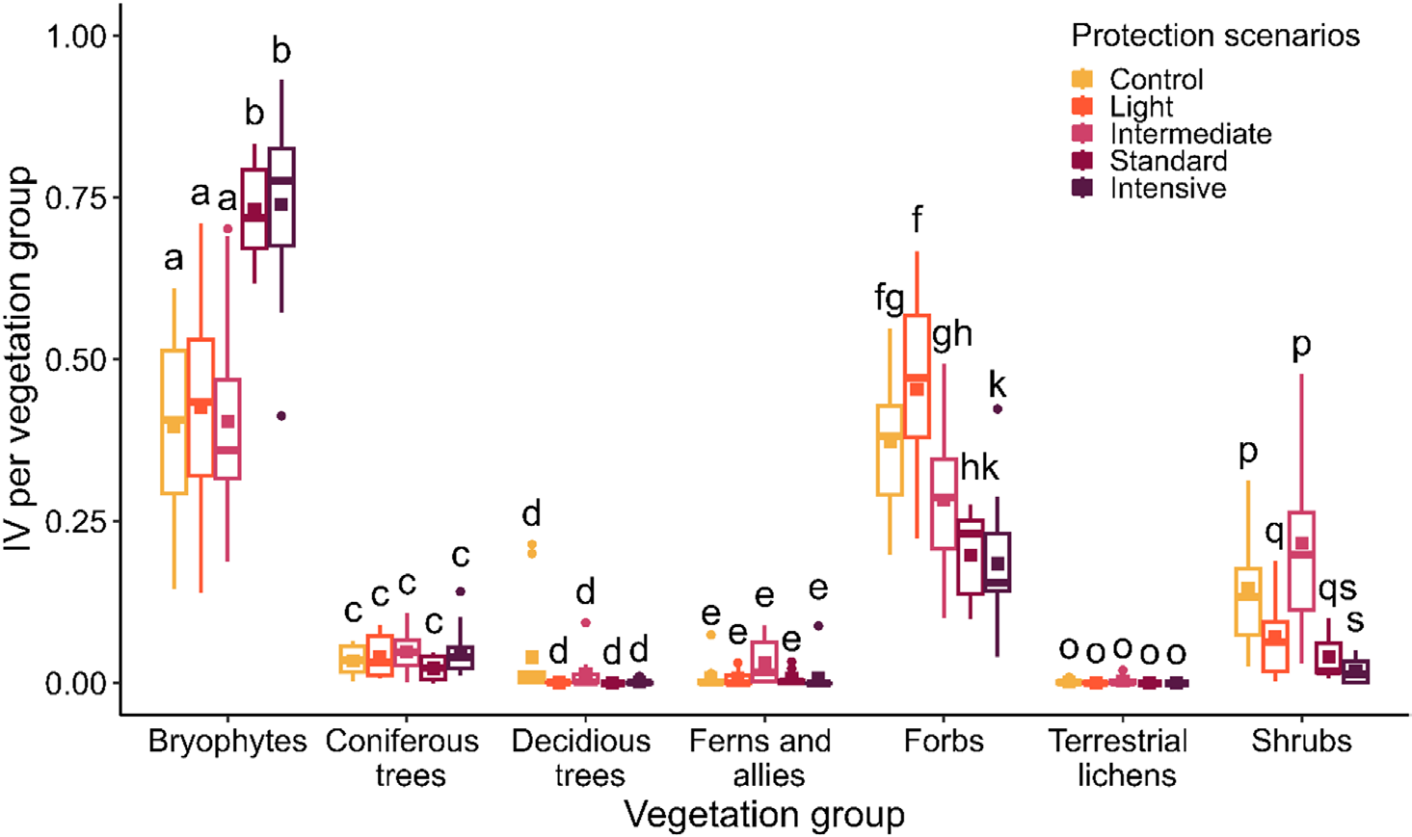
Influence of Btk protection scenarios that have been applied in northeastern Québec since 2007 on Importance Values of vegetation group calculated with 2022 data. Color square: Mean; bold horizontal line: Median; boxes: 25%–75% quartiles; whisker: Ranges; color dot: Outliers. Lower case letters indicate significant differences among protection scenarios within each plant life‐form group (*p* < 0.05).

We found a significant interaction between Btk spraying scenarios and plant life‐form groups (*F*_(24,364)_ = [12.533], *p* < 0.001), meaning that the IVs of plants among protection scenarios varied from one group to another. Btk spraying scenarios did not influence the IV of coniferous trees, deciduous trees, ferns/allies, and terrestrial lichens (Figure 4), indicating that these plant groups had the same relative importance in the communities regardless of the Btk spraying frequency. In general, forbs and shrubs had higher IVs on plots under control, light, or intermediate scenarios, while the opposite was found for bryophytes.

**FIGURE 5:**
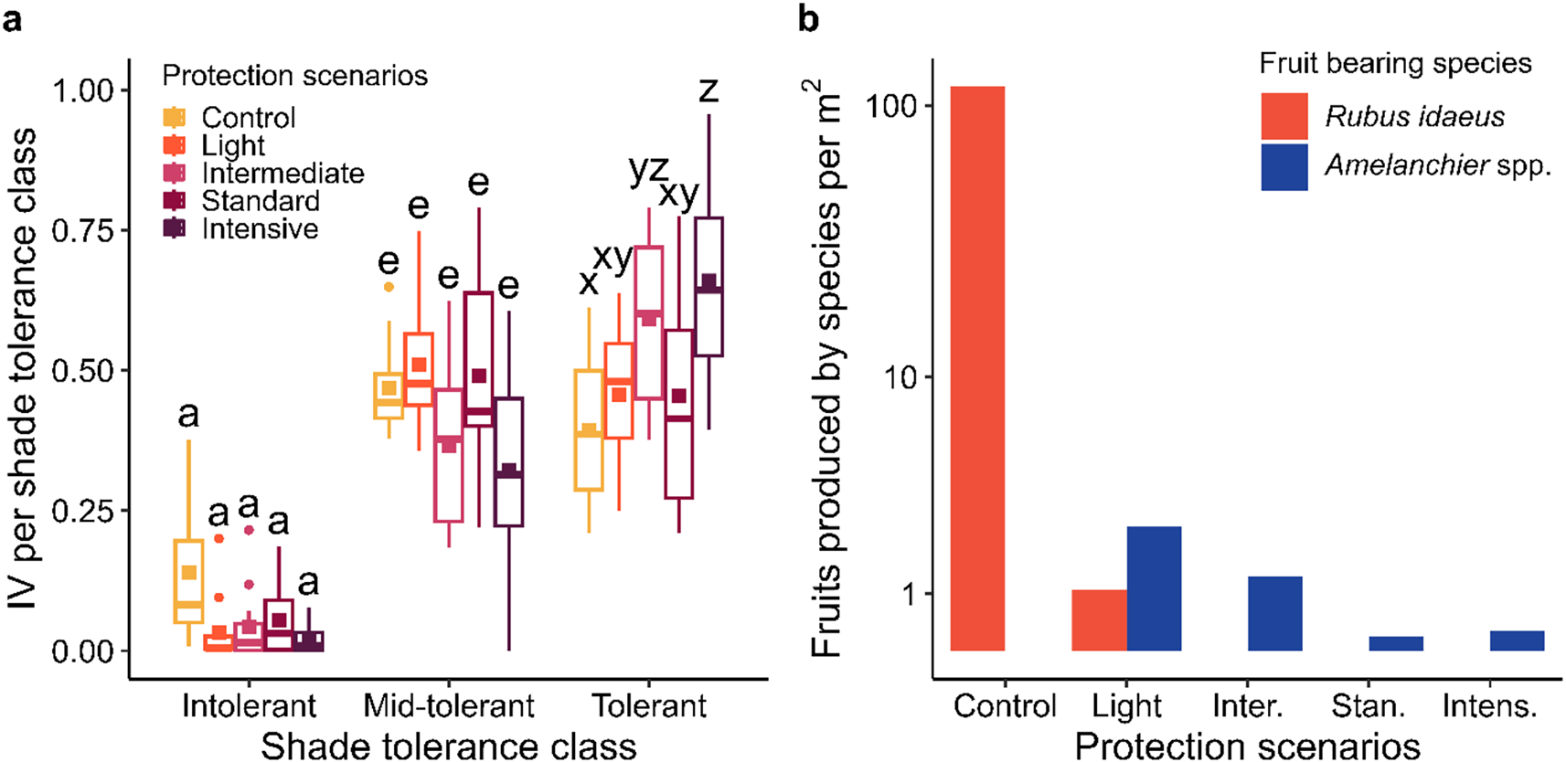
Influence of Btk protection scenarios that have been applied in northeastern Québec since 2007 on (a) shade tolerance of understory plant species and (b) total number of fruits produced per m^2^ per Btk protection scenario for two fruit bearing species according to 2022 inventories. Logarithmic scale is used on the y axis in (b). Color square: Mean; bold horizontal line: Median; boxes: 25%–75% quartiles; whisker: Ranges; color dot: Outliers. Lower case letters indicate significant differences among protection scenarios within each shade tolerance class (*p* < 0.05).

On average, shade and mid‐shade‐tolerant species were more abundant (percent cover of 51% and 43%, respectively) than shade‐intolerant species (5%) (Table S3). We found a significant interaction between Btk spraying scenarios and shade tolerance class (*F*_(8,156)_ = [7.053], *p* < 0.001), meaning that the IVs among scenarios varied from one shade tolerance class to another (Figure 5a). For instance, control plots were associated with the greatest IV of shade‐intolerant species, but the smallest IV of shade‐tolerant species, while the opposite was observed for plots under the intensive protection scenario. Furthermore, the IV of shade‐tolerant species in the latter scenario was significantly higher than in control, light, and standard plots and was also higher in plots of the intermediate protection scenario than in control plots.

Finally, we found more fruits per m^2^ in control plots than in any other protection scenario, especially *R. idaeus* fruits (117 fruits/m^2^ across control plots compared to 1.08 fruits/m^2^ across the light protection scenario; Figure 5b). *Amelanchier* spp. fruit production increased with decreasing Btk spraying frequency (ranging from 0.33 to 2.5 fruits/m^2^ produced in plots under intensive and light protection scenarios respectively), except for control plots where no *Amelanchier* spp. fruits were counted.

### 3.3 Relationship Between Btk Protection Scenarios and Beta Diversity

**FIGURE 6:**
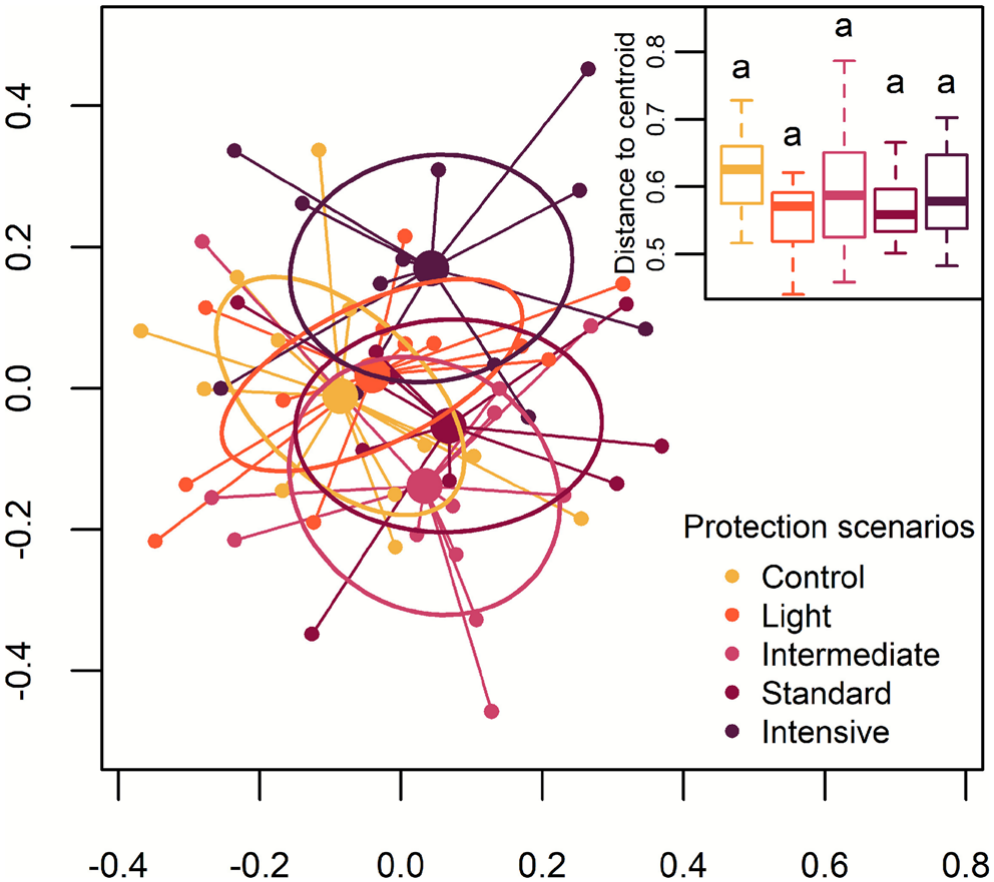
Principal coordinate analysis (PCoA) ordination showing compositional change in 2022 understory vegetation among Btk protection scenarios that have been applied in northeastern Québec since 2007. β‐diversity was measured as the multivariate dispersion of sites around their centroid (based on the Hellinger distance, represented in the top right boxplot) and was compared using tests for the multivariate homogeneity of group dispersions. Ellipses represent standard deviation.

We found no significant differences in beta diversity (site dispersion around their centroid) among Btk protection scenarios (*F*_(4,52)_ = [1.561], *p* = 0.197), but species composition (site centroid position) differed between protection scenarios (*F*_(4,52)_ = [1.820], *p* = 0.001) (Figure 6). Species composition was similar in control plots and in plots under the light protection scenario. Communities of intermediate, and particularly of the standard protection scenario were also similar to those in control plots and plots under the light protection scenario, while plots under the intensive protection scenario differed from the other protection scenarios. The spacing among intermediate, standard, and intensive centroids position indicates a species composition turnover. For instance, some species that were absent from plots under the intensive protection scenario were present in several control plots (e.g., *R. idaeus* and *Chamerion angustifolium* subsp. *angustifolium*) while other species that were uncommon in plots under the intensive protection scenario were more common in control plots (e.g., *Acer spicatum* , *Sorbus decora* and *S. americana* ).

## 4 Discussion

### 4.1 High Btk Treatment Frequency Leads to Higher Stand PAI

Consistent with other studies, we found that Btk aerial spraying effectively protected the canopy cover of *A. balsamea* ‐dominated stands (Fuentealba et al. 2015, 2019; Liu et al. 2020). In our experimental study, the PAI of plots under the intermediate and standard protection scenarios was equivalent to that of plots under the intensive protection scenario. This suggests that intermediate treatment frequency (i.e., spraying every 2 years) would be sufficient to prevent an increase in light availability in the understory and consequently reduce application costs. Although, the infested area in the study region is decreasing, the most recent data indicate that severe defoliation is likely to persist in the area (MRNF 2024). Given that the outbreak lasted 18 years so far, our results help fill a valuable gap in our knowledge of the long‐term effects of SBW control with Btk, as previous studies were conducted during the peak of the epidemic (Fuentealba et al. 2015; Liu et al. 2020).

### 4.2 Btk Treatments Affect Species Richness but Not Diversity

Our results indicate that understory species richness was higher at low than at high Btk spraying frequencies. The greater tree mortality and subsequent increase in resource availability (e.g., temperature, light, water, and nutrient availability) associated with lower levels of insect control likely facilitated the establishment of light‐demanding and nutrient‐demanding plant species, thereby fostering richness (Hart and Chen 2006). In the absence of disturbances in the understory layers, shade‐tolerant species that were already present before the increase in tree mortality may have persisted by occupying favorable microsites (de Grandpré and Bergeron 1997). The co‐existence of early‐ and late‐successional species could thus explain why sites under the control, light, and intermediate protection scenarios had higher species richness than under more intense scenarios (Table S3). Furthermore, the relatively slow changes in canopy conditions following SBW outbreaks and treatments, combined with regional species‐poor communities (de Grandpré et al. 2003), could also be related to similar beta diversity among the different protection scenarios. However, as the peak of understory beta diversity in boreal forests generally occurs 40 years after a stand‐replacing disturbance (Kumar et al. 2018), our results may not fully capture the relationship between richness and SBW control.

Although understory species richness was higher under low than under high Btk spraying frequencies, no significant differences were detected in the Shannon diversity index among protection scenarios. While species richness solely reflects the number of species present at a site, the Shannon diversity index integrates both species richness and their relative abundances (Whittaker et al. 2001). Discrepancies between richness and Shannon diversity often indicate changes in community structure, particularly in species evenness. Consistent with this interpretation, a posteriori analyses revealed that Pielou's evenness differed strongly among treatments (Kruskal–Wallis, *χ* ^2^ = 110.7, *p* < 0.001), with significantly lower evenness in the standard and intensive treatments compared to the control, light, and intermediate treatments, while no differences were detected among the latter three (Figure S3). This pattern suggests that in the standard and intensive treatments, a reduced number of species dominated the understory community ( *Cornus canadensis* in both treatments), leading to both lower richness and lower evenness. In contrast, the control, light, and intermediate treatments supported more species with more balanced relative abundances. Similar Shannon diversity values across treatments likely reflect compensatory effects between richness and evenness, with lower species richness and higher dominance in standard and intensive treatments offsetting higher richness but lower evenness in the other treatments.

### 4.3 Btk Treatments Affect Understory Community Composition

As we predicted, reducing spraying frequency led to changes in understory community composition associated with species turnover among life‐form and shade‐tolerance groups. Plots under the intensive protection scenarios were characterized by plant communities with a low proportion of shrubs and forbs, almost solely composed of shade‐tolerant and mid‐shade‐tolerant species, and with fruit‐bearing species producing virtually no fruit. In contrast, control plots were dominated by forbs and shrubs (mainly deciduous and fruit‐bearing shrubs) and had a more balanced proportion of the three shade‐tolerance classes and a high abundance of fruits. Similar shifts in species composition, including increased deciduous regeneration and fruit‐bearing species cover with SBW epidemic severity, were previously observed throughout the Quebec boreal forest (Chagnon et al. 2022). The observed changes in understory composition may persist through time, as increased cover of deciduous regeneration with SBW epidemics can transform a coniferous stand into a deciduous stand which allows more light transmission to the understory, which in turn further promotes light‐ and nutrient‐demanding species (Bouchard et al. 2006; Chagnon et al. 2022; Fourrier et al. 2015; Sánchez‐Pinillos et al. 2019).

## 5 Conclusions and Further Studies

Overall, the results of our experimental study show that reducing the spraying frequency increased light availability in the understory, leading to higher vascular plant richness, increased cover of shrubs, forbs, and shade‐intolerant species, and a greater number of fruits produced by fleshy‐fruit bearing plants, while arboreal lichen biomass remained unchanged. Although annual spraying (intensive scenario) most effectively limits SBW‐induced defoliation, such an approach may not be necessary to maintain canopy closure. In this context, the intermediate spraying regime, based on biennial applications, appears to offer a balanced alternative by maintaining foliage during SBW infestation while limiting shifts of the understory toward early‐successional vegetation. Given the scale and long‐term nature of the experimental design, these conclusions are based on a level of replication that we consider sufficient to detect ecologically meaningful vegetation responses; however, we acknowledge that a larger experimental design might have allowed the detection of more subtle differences among treatments.

In this study, we focused exclusively on the effects of spruce budworm (SBW) control on understory vegetation in boreal forests. However, in our study region, these results may also have implications for woodland caribou management, a threatened species in Canada (COSEWIC 2002). Btk spraying frequency may influence vegetation changes that may be associated with altered competitor–predator–prey dynamics in boreal woodland caribou habitats (Labadie et al. 2021). Nevertheless, the direct effects of these vegetation changes on caribou populations remain to be evaluated. Future studies should therefore integrate a nutritional ecology perspective (e.g., plant biomass and forage quality) with habitat selection data.

## Author Contributions

**Mathilde Robitaille:** conceptualization (equal), data curation (lead), formal analysis (lead), investigation (equal), methodology (equal), writing – original draft (lead). **David Pothier:** conceptualization (equal), funding acquisition (lead), methodology (equal), supervision (equal), writing – review and editing (equal). **Stéphanie Pellerin:** conceptualization (equal), methodology (equal), supervision (equal), writing – review and editing (equal).

## Funding

This research was funded by Ministère des Ressources naturelles et des Forêts of Québec (grant number: 20211423619‐02). Fonds de recherche du Québec‐Nature et technologies and Conseil de recherches en sciences naturelles et en génie du Canada provided scholarships to M. Robitaille.

## Conflicts of Interest

The authors declare no conflicts of interest.

## Supporting information


**Data S1:** ece373188‐sup‐0001‐Supinfo.docx.

## Data Availability

Data and scripts are available at: https://github.com/MathildeRobitaille/Btk_SBW.
