# Supplementary material for: Pairing Spruce Budworm Control and Minimal Understory Perturbations: Effects of Btk Spraying Frequency in Boreal Forests
Source: Ecol Evol. 2026 Feb 27;16(3):e73188. doi: 10.1002/ece3.73188 (PMC12949341; doi:10.1002/ece3.73188)
Supplement: Supplementary file 1 — Data S1: ece373188‐sup‐0001‐Supinfo.docx. [file ECE3-16-e73188-s001.docx]

**Supplementary materials**

**
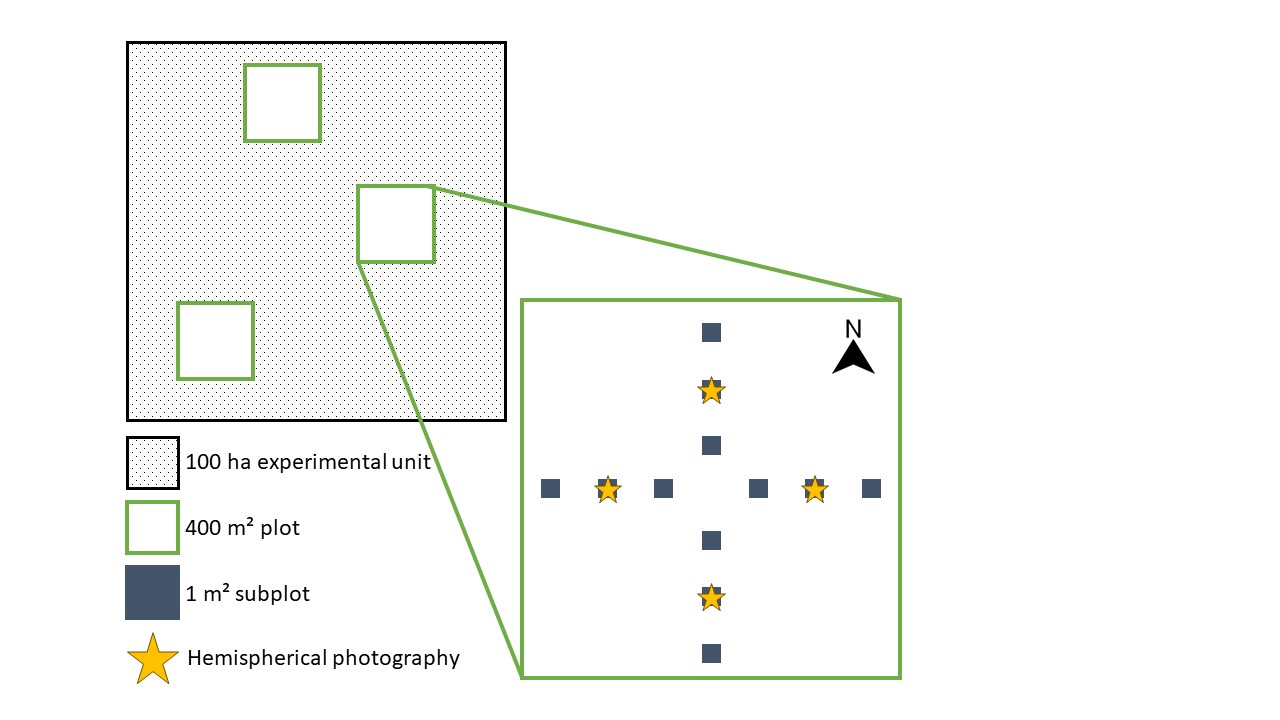
**

**Fig. S1.** Study design for assessing the impact of Btk spraying scenarios on richness and diversity of understory vegetation in boreal forests of Quebec, Canada. The 100-ha experimental units correspond to the area in which the Btk spray treatments were applied. The 400-m^2^ plots correspond to the area where all the samplings were conducted. The 1-m^2^ subplots were used to sample understory vegetation and fleshy-fruits.

**
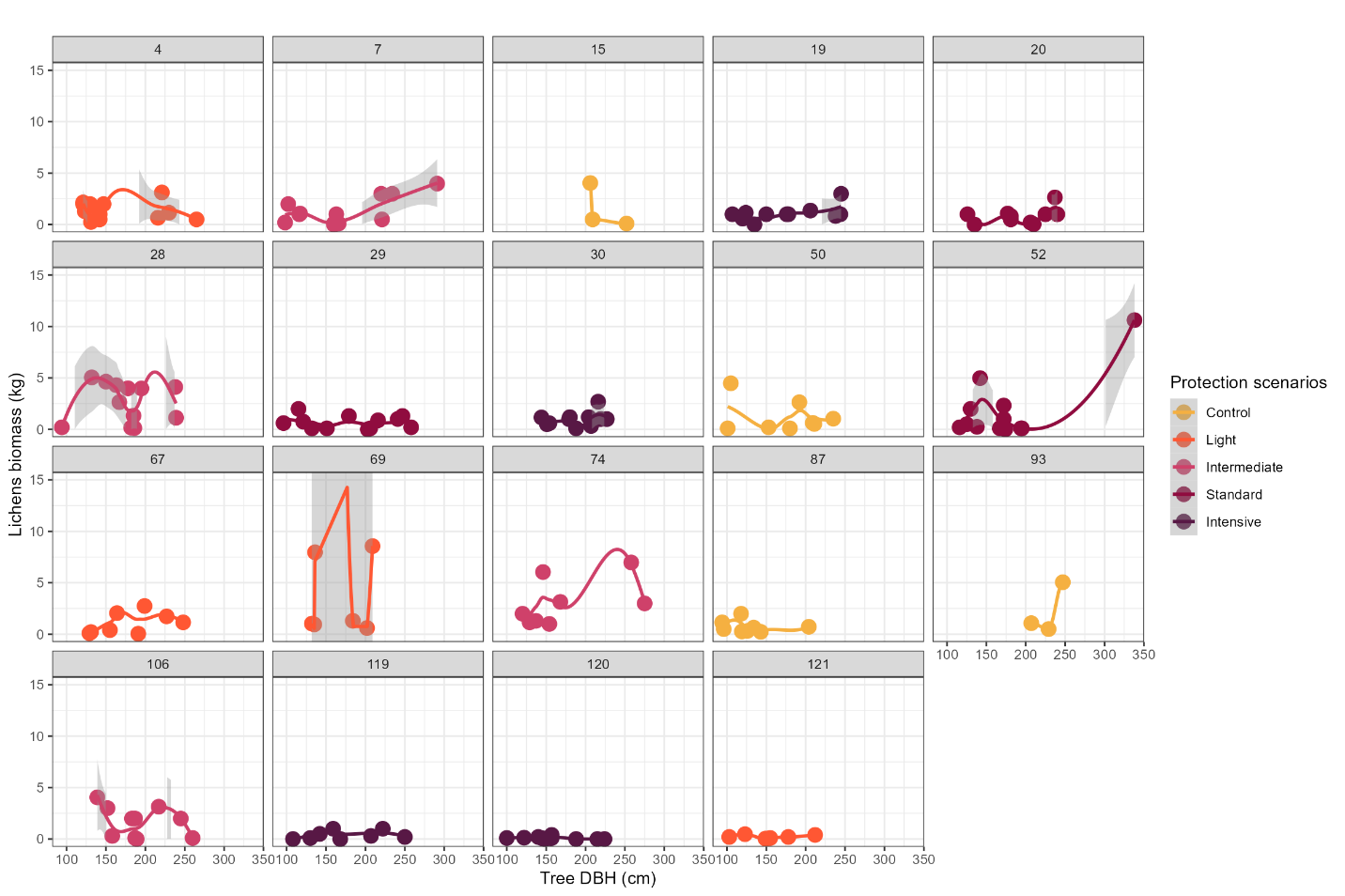
**

**Fig. S2.** Relation between lichen biomass and tree diameter at breast height (DBH) measured in 2022 in each experimental units from an experimental study design established in 2017 for assessing the impact of Btk spraying scenarios on richness and diversity of understory vegetation in boreal forests of Quebec, Canada.

**
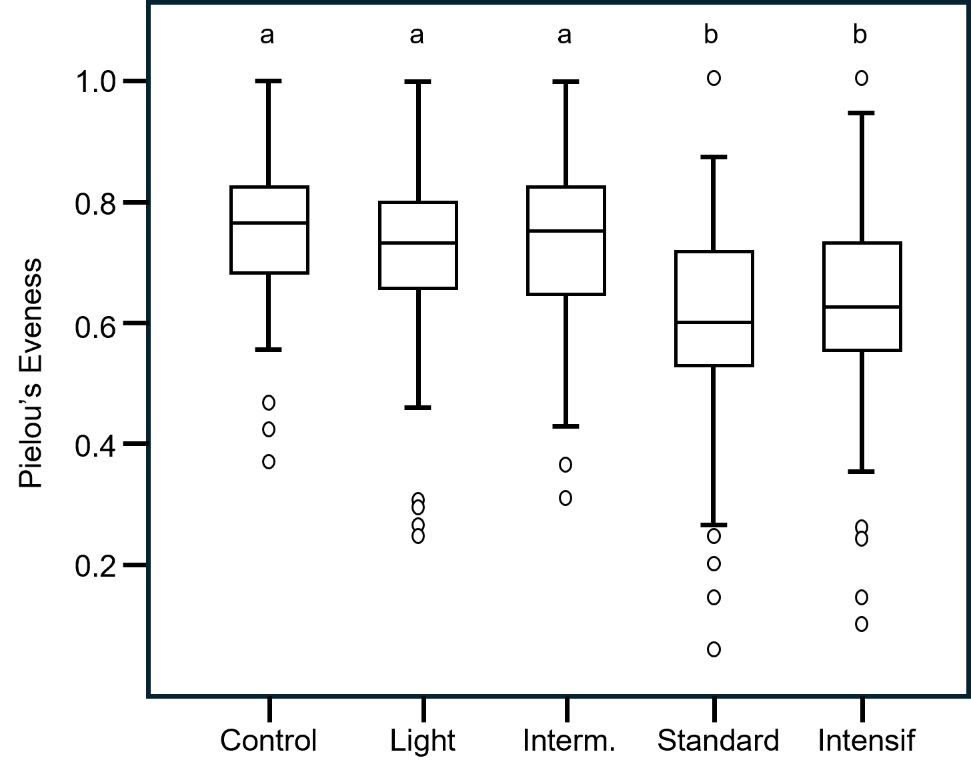
**

**Fig. S3.** Influence of Btk protection scenarios that have been applied in northeastern Québec since 2007 on Pielou’s Eveness index calculated with 2022 data. mean; bold horizontal line: median; boxes: 25‑75 % quartiles; whisker: ranges; dot: outliers. Differences among treatments were first assessed using a Kruskal-Wallis test. When significant, pairwise differences were evaluated using Mann–Whitney U tests with Holm correction to account for multiple comparisons. Different lower-case letters indicate a significant difference between protection scenarios (*p* < 0.05).

**Table S1.** List of all models constructed for the statistical analyses used to determine the effects of a gradient of five Btk aerial spraying frequencies on SBW-induced tree defoliation, understory vegetation, and arboreal lichen biomass using an experimental design established in 2007. We presented the specific question that each model is seeking to answer, the X and Y variables (i.e., independent and dependent variables, respectively), the random effect as well as the transformation used when needed.

| **Question** | **X variable** | **Y variable** | **Random effect** | **Transformation** |
| --- | --- | --- | --- | --- |
| Are there differences in stand PAI between Btk protection scenarios? | Btk protection scenarios | PAI (m² m^-2^) | (1\|PlotID) | Log |
| Are there differences in lichen biomass between Btk protection scenarios? | Btk protection scenarios | Lichens biomass (kg/ha) | (1\|PlotID) | Log |
| Are there differences in species richness between Btk protection scenarios? | Btk protection scenarios | Species richness | (1\|PlotID) | None |
| Are there differences in Shannon’s diversity index between Btk protection scenarios? | Btk protection scenarios | Shannon Weaver Index | (1\|PlotID) | None |
| Are there differences in the presence of shade tolerance groups between Btk protection scenarios? | Btk protection scenarios * Shade tolerance group | IV of shade tolerance group | (1\|PlotID) | None |
| Are there differences in the presence of life form groups between Btk protection scenarios? | Btk protection scenarios * life form group | IV of life form group | (1\|PlotID) | Arcsin |

**Table S2.** List bioclimatic and environmental variables evaluated and tested in statistical analyses used to determine the effects of a gradient of five Btk aerial spraying frequencies on SBW-induced tree defoliation, understory vegetation, and arboreal lichen biomass using an experimental design established in 2007. For bioclimatic variables, the 20-year monthly average during the growth period (data May to October) from 2001 to 2021 was calculated. To reduce the number of variables and avoid multicollinearity, only variables in grey were selected for the models.

| **Variable description** | **Unit** |
| --- | --- |
| Lowest temperature recorded of the 20 years surveyed | °C |
| Highest temperature recorded of the 20 years surveyed | °C |
| Mean of the minimal temperature recorded each month | °C |
| Mean of the maximal temperature recorded each month | °C |
| Mean yearly temperature | °C |
| Mean yearly precipitation as rain | mm |
| Mean of yearly total precipitation as rain | mm |
| Relative humidity | % |
| Mean of the number of yearly rainy days | Days |
| Highest number of consecutive days with no rainfall of the 20 years surveyed | Days |
| Degree days | °C $\times$ days |
| Nearest distance between each plot and the coast. | Km |
| Surface deposit on each plot | Till, Glaciofluvial, Raised beach |
| Topographic wetness index | 1 to 18 |
| Severity and starting date of SBW epidemics declared on each plot | Light or Severe |
| Time since the beginning of the SBW epidemics | Julian day |
| Distance between each plot and the closest fire within a 5-km buffer. | Km |
| Time since the last closest fire | Julian day |
| Distance between each plot and the closest logging area within a 5-km buffer. | Km |
| Time since the last closest logging | Julian day |
| Distance of each plot to the closest road | M |
| Road density in a 1km² radius of the plot | Km/km^2^ |

**Table S3.** List of plant taxa that were sampled. The average cover of each species (%) in each Btk protection scenario is presented as well as the shade tolerance level for vascular species (T: shade tolerant, M: mid-shade tolerant, I: shade intolerant, and NA: information not available).

| **Species** | **Control** | **Light** | **Intermediate** | **Standard** | **Intensive** | **Shade Tolerance** |
| --- | --- | --- | --- | --- | --- | --- |
| **Coniferous trees (< 1.5 m)** | | | | | | |
| *Abies balsamea* | 4.7 | 6.9 | 6.1 | 2.2 | 4.2 | T |
| *Picea glauca* | 0.3 | 0.1 | 0.1 | 0.0* | 0.0 | T |
| *Picea mariana* | 0.6 | 0.9 | 0.3 | 0.2 | 0.6 | T |
| **Decideous trees (< 1.5 m)** |  |  |  |  |  |  |
| *Acer rubrum* | 2.5 | 0.0 | 0.2 | 0.0 | 0.0 | M |
| *Betula alleghaniensis* |  |  | 0.0 |  |  | M |
| *Betula papyrifera* | 0.8 | 0.1 | 1.0 | 0.0 | 0.1 | I |
| *Populus balsamifera* |  | 0.0 |  |  |  | I |
| *Populus tremuloides* | 0.1 |  |  | 0.0 |  | I |
| **Shrubs** |  |  |  |  |  |  |
| *Acer spicatum* | 1.1 | 2.2 | 5.3 | 0.4 | 0.4 | T |
| *Alnus incana* subsp. *rugosa* | 0.0 | 0.4 | 0.0 |  | 0.0 | I |
| *Amelanchier* spp. | 0.5 | 4.8 | 4.2 | 1.9 | 0.5 | NA |
| *Cornus alternifolia* |  | 0.1 | 0.2 |  |  | T |
| *Diervilla lonicera* | 1.4 | 0.3 | 2.8 | 0.1 |  | M |
| *Kalmia angustifolia* | 0.4 | 0.3 | 0.0 | 0.9 |  | I |
| *Prunus pensylvanica* | 0.4 |  |  |  |  | I |
| *Ribes glandulosum* | 0.4 |  |  |  |  | M |
| *Ribes lacustre* | 0.1 |  |  |  |  | T |
| *Ribes triste* | 0.2 |  |  |  |  | T |
| *Rubus idaeus* | 13.4 | 1.5 |  |  |  | I |
| *Sambucus racemosa* | 0.1 | 0.4 |  |  |  | M |
| *Sorbus americana* | 0.6 | 0.6 | 0.2 | 0.0 | 0.0 | M |
| *Sorbus decora* | 0.8 | 0.3 | 0.9 | 0.2 | 0.0 | M |
| *Taxus canadensis* | 1.0 | 1.2 | 10.1 |  |  | T |
| *Vaccinium angustifolium* | 1.0 | 0.5 | 1.3 | 0.6 | 0.5 | I |
| *Vaccinium myrtilloides* |  | 0.3 | 0.0 | 0.1 | 0.3 | M |
| *Viburnum edule* |  | 0.5 | 0.3 |  |  | M |
| *Viburnum cassinoides* | 0.3 |  |  |  |  | T |

* A value of 0.0 indicates a mean cover smaller than 0.1

**Table S1.** Continued.

| **Species** | **Control** | **Light** | **Intermediate** | **Standard** | **Intensive** | **Shade Tolerance** |
| --- | --- | --- | --- | --- | --- | --- |
| **Forbs** |  |  |  |  |  |  |
| *Anaphalis margaritacea* | 0.0 |  |  |  |  | I |
| *Aralia nudicaulis* | 1.4 | 7.8 | 2.5 | 0.3 | 0.7 | T |
| *Chamerion angustifolium* | 0.5 | 0.3 |  |  |  | I |
| *Clintonia borealis* | 3.7 | 10.4 | 7.9 | 2.3 | 1.6 | M |
| *Coptis trifolia* | 1.7 | 4.0 | 4.4 | 3.2 | 1.8 | T |
| *Cornus canadensis* | 23.3 | 25.6 | 8.7 | 7.4 | 4.4 | M |
| *Cypripedium acaule* | 0.1 |  |  |  |  | T |
| *Eurybia macrophylla* |  | 0.0 | 0.3 | 0.0 |  | M |
| *Epigaea repens* |  |  |  | 0.3 |  | M |
| *Galium triflorum* | 0.4 |  |  |  |  | T |
| *Gaultheria hispidula* | 3.2 | 1.4 | 0.4 | 0.8 | 0.7 | T |
| *Goodyera tesselata* | 0.0 | 0.0 | 0.0 |  | 0.1 | T |
| *Goodyera repens* | 0.2 | 0.1 | 0.1 | 0.1 | 0.2 | T |
| *Linnaea borealis* | 10.3 | 12.8 | 5.1 | 1.0 | 2.3 | M |
| *Lysimachia borealis* | 2.6 | 4.9 | 2.4 | 0.2 | 0.2 | T |
| *Maianthemum canadense* | 7.1 | 11.3 | 4.1 | 2.1 | 3.1 | T |
| *Mitella nuda* |  |  | 0.7 |  |  | T |
| *Moneses uniflora* | 0.1 | 0.3 | 0.1 | 0.0 | 0.1 | M |
| *Monotropa uniflora* |  |  |  | 0.0 | 0.0 | T |
| *Nabalus* spp*.* |  |  | 0.0 |  |  | T |
| *Neottia cordata* |  | 0.0 |  |  |  | T |
| *Oclemena acuminata* |  |  | 0.0 |  |  | T |
| *Orthilia secunda* | 0.5 | 0.6 | 0.7 | 0.7 | 0.8 | T |
| *Oxalis montana* | 5.5 | 5.6 | 1.8 | 0.9 | 1.5 | T |
| *Rubus pubescens* | 0.0 | 1.0 | 0.0 |  |  | T |
| *Streptopus amplexifolius* |  | 0.0 | 0.1 |  |  | M |
| *Trillium undulatum* |  |  | 0.1 | 0.1 |  | T |
| *Viola* spp. | 0.0 | 0.0 |  |  |  | T |
| **Grass** |  |  |  |  |  |  |
| *Carex* spp. | 0.0 | 0.5 |  | 0.0 |  | NA |

**Table S1.** Continued.

| **Species** | **Control** | **Light** | **Intermediate** | **Standard** | **Intensive** | **Shade Tolerance** |
| --- | --- | --- | --- | --- | --- | --- |
| **Ferns and allies** |  |  |  |  |  |  |
| *Athyrium filix.femina* |  | 4.4 |  |  |  | T |
| *Claytosmunda claytoniana* |  |  |  | 0.4 |  | M |
| *Dendrolycopodium dendroideum* | 0.0 | 0.1 | 0.2 | 0.1 |  | T |
| *Dryopteris cristata* | 0.4 |  | 0.0 |  |  | T |
| *Dryopteris intermedia* | 0.0 | 0.0 | 0.3 |  |  | T |
| *Equisetum sylvaticum* | 0.0 | 0.0 |  |  |  | T |
| *Gymnocarpium dryopteris* | 0.1 | 0.7 | 0.4 |  |  | T |
| *Huperzia lucidula* |  |  | 0.0 |  |  | T |
| *Phegopteris connectilis* | 0.0 | 0.2 |  |  |  | T |
| *Pteridium aquilinum* | 0.5 |  | 2.5 | 0.3 | 0.0 | M |
| *Spinulum annotinum* | 0.0 |  | 0.1 | 0.0 |  | T |
| **Bryophytes** |  |  |  |  |  |  |
| *Bazzania trilobata* | 0.5 | 0.0 |  |  | 0.1 |  |
| *Dicranum* spp. | 4.8 | 3.8 | 8.8 | 6.2 | 7.7 |  |
| *Hylocomium splendens* | 19.3 | 16.0 | 3.8 | 14.0 | 17.7 |  |
| *Pleuzorium schreberi* | 27.6 | 40.8 | 33.3 | 47.9 | 43.3 |  |
| *Polytrichum* spp. |  |  |  | 0.0 |  |  |
| *Ptilium crista castrensis* | 5.9 | 5.7 | 5.3 | 3.9 | 1.9 |  |
| *Rhytidiadelphus triquetrus* | 0.1 |  | 0.7 |  |  |  |
| *Sphagnum* spp. | 0.2 | 1.5 | 0.0 | 0.8 |  |  |
| **Terrestrious lichens** |  |  |  |  |  |  |
| *Cladonia* spp. |  |  | 0.0 |  |  |  |
| *Cladonia coniocraea* | 0.2 | 0.1 | 0.2 | 0.0 |  |  |
| *Cladonia rangiferina* | 0.1 | 0.0 | 0.4 |  |  |  |
